# Effect of Spaceflight on the Circadian Rhythm, Lifespan and Gene Expression of *Drosophila melanogaster*

**DOI:** 10.1371/journal.pone.0121600

**Published:** 2015-03-23

**Authors:** Lingling Ma, Jun Ma, Kanyan Xu

**Affiliations:** Laboratory of Space Microbiology, Shenzhou Space Biotechnology Group, China Academy of Space Technology, Beijing, China; University of Lübeck, GERMANY

## Abstract

Space travelers are reported to experience circadian rhythm disruption during spaceflight. However, how the space environment affects circadian rhythm is yet to be determined. The major focus of this study was to investigate the effect of spaceflight on the *Drosophila* circadian clock at both the behavioral and molecular level. We used China’s Shenzhou-9 spaceship to carry *Drosophila*. After 13 days of spaceflight, behavior tests showed that the flies maintained normal locomotor activity rhythm and sleep pattern. The expression level and rhythm of major clock genes were also unaffected. However, expression profiling showed differentially regulated output genes of the circadian clock system between space flown and control flies, suggesting that spaceflight affected the circadian output pathway. We also investigated other physiological effects of spaceflight such as lipid metabolism and lifespan, and searched genes significantly affected by spaceflight using microarray analysis. These results provide new information on the effects of spaceflight on circadian rhythm, lipid metabolism and lifespan. Furthermore, we showed that studying the effect of spaceflight on gene expression using samples collected at different Zeitgeber time could obtain different results, suggesting the importance of appropriate sampling procedures in studies on the effects of spaceflight.

## Introduction

Spaceflight has complex effects on the physiology of organisms from Earth. Major phenotypic changes include bone loss, muscle atrophy, altered energy metabolism, increased aging, circadian disruption, and sleep loss [1–5]. Among the physiological functions affected by spaceflight, the circadian clock system plays an important role in regulating various aspects of animal behavior and physiology. Circadian clock malfunctions can lead to a variety of pathological conditions including sleep disorders, cardiovascular disorders, mental depression, metabolic syndromes, and inflammation [6–9]. Due to the important physiological functions of the circadian clock, its alteration during spaceflight could lead to serious health consequences. It is possible that some physiological phenotypic changes observed in astronauts and space flown animals may be caused by alteration of the circadian system. For example, various studies have reported that sleep, a well-known clock regulated behavior, changed in both duration and structure in space [10–14], and misalignment of sleep and circadian rhythm impaired the health, alertness and performance of astronauts [15]. Thus, understanding the influence of the space environment on the circadian clock system is of great importance for long-duration space exploration.

Although the potential impacts of space on the circadian clock system of animals and human beings were recognized shortly after spaceflight began, and the first report on its effect on *Neurospora* circadian rhythm was published in 1984 [16], little progress has been made during the last 30 years. One important reason is the complexity of the space environment. During spaceflight, many environmental factors are changed dramatically, including a 24-h light-dark cycle, a gravitational force of 1 G, various stress stimuli, and altered feeding habits. All have been reported as possible Zeitgebers of the circadian clock. Thus, the altered circadian rhythm observed in both astronauts and test animals in space could be caused by the combined effects of environmental change. This environmental complexity has impeded research on the mechanism of how the space environment influences the circadian clock system, which still remains largely unknown.

The first question is whether and how gravity changes affect the circadian clock system. Unlike light, which is well accepted as the strongest Zeitgeber of the circadian clock, the relationship between gravity and the circadian clock is less clear. Several reports have shown that altered gravity can affect the amplitude of circadian rhythms in species from unicellular organisms to humans [17–21], and gravity changes can induce both phase-shift and synchronization of the circadian pacemaker [22, 23]. However, these differences were not as severe as those caused by light changes, which suggests that gravity may be a weak Zeitgeber of the circadian clock. Since no proof exists at the molecular level to confirm that gravity changes affect the rhythm of major clock genes in a specific organ, the role of gravity as a circadian Zeitgeber remains controversial.

Another question about the mechanism by which spaceflight affects the circadian clock is whether there is any circadian change at the molecular level. Although investigations on circadian rhythm changes during spaceflight have continued for more than 30 years, most published results have focused on physiological or behavioral research, such as body temperature rhythm [10, 24,25], free-running activity rhythm [26, 27], and blood pressure rhythm [28, 29]. There are few reports studying the effect of spaceflight on the expression of major circadian clock genes and circadian output genes at the molecular level. Thus, the possible mechanisms underlying circadian changes during spaceflight are still largely unknown.

*Drosophila* is a powerful model for circadian rhythm and sleep studies, exhibiting similar circadian clock and sleep regulation mechanisms as mammals [30, 31]. *Drosophila* is also widely used for studying the biological effect of the space environment [32–34]. Due to its small size and simple breeding conditions, *Drosophila* can be loaded onto spaceships in large numbers even when astronaut participation is unlikely. In this study, we used the Shenzhou-9 spaceship to carry *Drosophila* samples and performed various experiments to investigate the effects of spaceflight on circadian rhythm as well as on other physiological phenotypes.

## Materials and Methods

### Fly line and experimental conditions

Wild type male Canton-S flies were used in all experiments. Flies were grown on regular *Drosophila* food purchased from the Qingdao Hope Bio-Technology Company (China). Five hundred and forty male flies (1–3 days old) were collected and divided into three groups. Space flown group flies were sent into space, the lab control group (control-2) was maintained in an incubator in our lab, while the handling control group (control-1) underwent the same traveling processes as space flown flies before launching and a simulated launch process on June 6, 2012. Both control-1 and control-2 flies were used as ground controls. For each group, 180 flies were put into 10 regular food vials (15–20 flies in each vial), which were transferred into a travel box specifically designed for this study. An LED light with an adjustable on/off mode powered by a lithium battery was placed in the travel box to maintain a 12 h:12 h light/dark cycle (LD) during spaceflight, with the light-on time set at 08:00 every day (GMT+8:00). Following instructions from the Shenzhou-9 spaceship project group, the travel box with space flown flies was sent to the China Jiuquan satellite launch site 10 days before the launch, where the box was put into the return cabin of the spaceship. The spaceship was launched on June 6, 2012, and stayed in orbit for 13 days. During the pre-launch and entire spaceflight period, the average temperature in both the return cabin and the incubator containing the two ground control boxes was 21 ± 3°C and the relative humidity was 35–60% (data from Shenzhou-9 spaceship project group).

### Fly recovery and weight measurement

After the return cabin landed (10:03 on June 29, 2012), the travel box with space flown flies was brought back to the lab. All three travel boxes were opened at 10:00 on July 1, 2012, which was 48 h after the landing. Dead flies were excluded from sample collection and the surviving flies were anesthetized by carbon dioxide and weighed using an electronic balance. After weight measurement, the flies for behavioral and lifespan testing experiment were put into locomotor activity tubes; the remaining flies were evenly divided into six groups and were maintained in fresh food vials in LD conditions with the same phase as in the travel box before sample collection. The first fly sample for the microarray and metabolic experiments was collected at 12:00, about 50 h after landing, and since the light-on time was set at 08:00, the Zeitgeber time of our first sample was ZT4. About 40% of space flown flies survived the spaceflight process, while the survival rates for control-1 and control-2 flies were 60% and 62%, respectively.

### Behavior and lifespan tests

We used 15 space flown, 31 control-1 and 32 control-2 flies for behavioral and lifespan testing. Flies were first loaded into locomotor activity monitoring tubes containing 5% sucrose/1% agar food and incubated under LD conditions with the same phase as in the travel box for 5 days. After that, flies were transferred and maintained in constant darkness (DD) until all flies died. Every 5 days, flies were transferred into a new monitoring tube with fresh food in order to maintain the health of the flies during the lifespan test. Fly locomotor activity was monitored using a DAM system (Trikinetics, USA); total activity was calculated as the total bout number recorded by the DAM system; locomotor activity was analyzed using FaasX and Clocklab2 software (USA); sleep patterns and parameters were analyzed using Pysolo 0.9 software (USA), with sleep defined as an interval of 5 min or more of behavioral immobility; fly mortality was defined by the cessation of locomotor activity.

### Fly sample collection and total RNA extraction

Flies for microarray and metabolic testing were incubated under LD conditions before collection. Samples were collected every 4 h, starting from ZT4, for a total of six time points. At each time point, 10–15 flies were killed on dry ice, fly heads and bodies were separated and stored in liquid nitrogen. Fly bodies were used for TAG and glycogen assays, and total RNA from fly heads was used for microarray and quantitative real time PCR (qPCR) experiments.

To collect total RNA, fly heads at each time point were divided into two parallel groups (at least five fly heads per group). Total RNA was extracted using Trizol reagent (Invitrogen, USA), and RNA quantity and quality were determined by Nanodrop 2000 (Thermo, USA) and electrophoresis.

### Microarray and data analysis

For each fly group, 100 ng of total RNA was first converted to cDNA, then an overnight *in vitro* transcription reaction was performed to generate a pool of cRNA carrying a biotin tag using a GeneChip3’ IVT Express Kit (AmbionInc., USA). The labelled cRNA samples were hybridized with *Drosophila* Genome 2.0 Array chips (Affymetrix, USA) following the protocol provided by the manufacturer. Data were acquired using a 7G GeneChip Scanner 3000 and processed by Expression Console software (Affymetrix, USA) using the RMA algorithm. Genes showing circadian expression patterns were identified using Short Time-series Expression Miner (STEM) software [35].

### Primer Design and real-time PCR

For the qPCR experiment, the mRNA levels of different genes were measured using SYBR Green PCR mix (Applied Biosystems, USA) in an ABI 7500 Sequence Detection System (Applied Biosystems). The following primers were designed using Beacon Designer and DNAMAN software: for *tim* mRNA: 5’ primer, CACTTCCGCAACAACAGAGT; 3’ primer, ACTCCGCAGGGTCAGTTTAA; for *vri* mRNA: 5’ primer, CGGCTATGGAGATGGAATGATG; 3’ primer, GCTCTCGTCCTTCTTGTTGTC; for *mus*209 mRNA: 5’primer, CAAGCCACCATCCTGAAGAAG; 3’primer, GCGAGACAAGCGACACAT; for *ilp*3 mRNA: 5’primer, AACGCAATGACCAAGAGAACT; 3’primer, TTGAGCATCTGAACCGAACTATC; for *kif*3c mRNA: 5’primer, CGCACTTAGGCACACCAA; 3’primer, TCCGCTCTCTCGCATTCT; for *rp*49 mRNA: 5’primer, CCGCTTCAAGGGACAGTATC; 3’primer, ATCTCGCCGCAGTAAACG. The level of *rp*49 mRNA was used as a control for the total RNA content in each sample. The values of other genes were normalized to those of *rp*49.

### TAG and glycogen measurement

Fly bodies were used for TAG and glycogen level tests. For each sample, fly bodies from all six time points were pooled together and divided into three parallel groups. After being washed twice using PBS, fly bodies were homogenized in homogenization buffer (20 μl per fly body), which was included in the Tissue triglyceride assay kit E1003-2 (Applygen Technologies Inc.). Protein concentration determination was performed using a BCA protein assay kit (Beyotime Institute of Biotechnology, China). Glycogen levels were measured using a glycogen assay kit KA0861 (Abnova). Protein concentrations were used to normalize glycogen and TAG levels in different samples.

## Results and Discussion

### Locomotor activity rhythm and sleep

**Fig 1:**
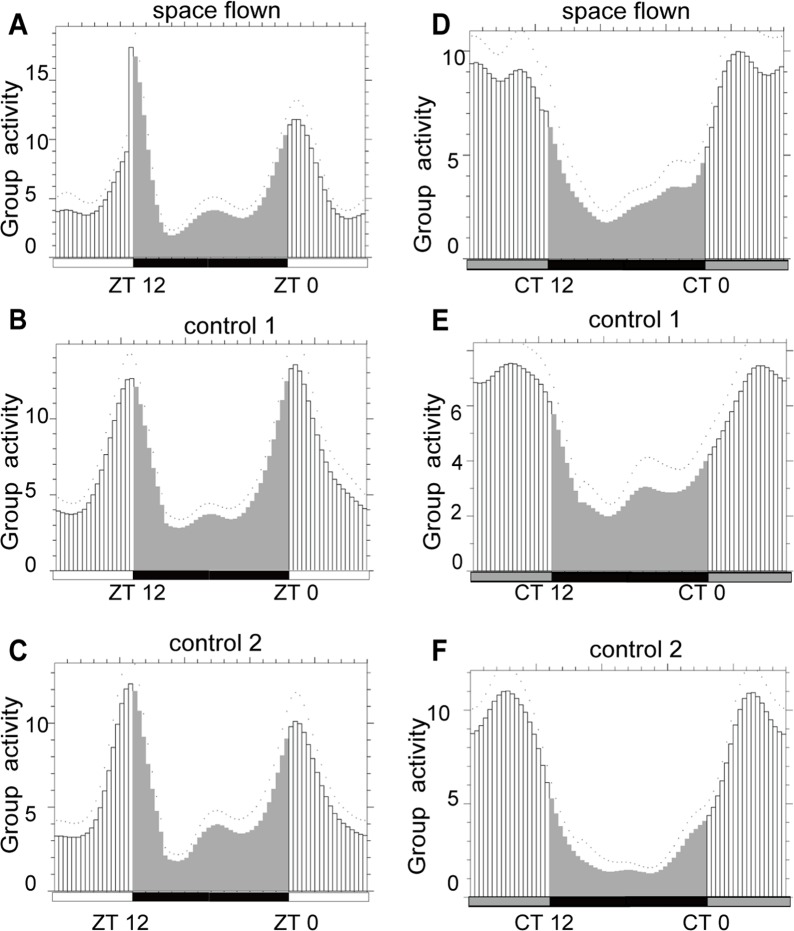
FaasX analysis showed that Drosophila locomotor activity rhythm was not changed after spaceflight. Space flown flies exhibited similar locomotor activity rhythm as the two control groups under both LD (A-C) and DD (D-F) conditions. White and black bars stand for daytime and nighttime, grey bar stands for subjective daytime in DD. Group activity was calculated by mean of five days. Two-way ANOVA was applied to the activity data for space flown and control flies at different Zeitgeber time, and the factor of treatment (space flown/control) was not significant by the conventional standard (p < 0.05).

To evaluate whether there were any changes in *Drosophila* locomotor activity rhythm post spaceflight, we compared the locomotor activity rhythms of space flown flies and the two control groups in both LD and DD conditions. Locomotor activity was monitored immediately after the space flown flies returned to the lab. We used both FaasX and Clocklab2 to analyze *Drosophila* activity rhythm. FaasX analysis showed that space flown flies exhibited normal locomotor activity rhythms as a group in both LD and DD conditions when compared with those of the two control groups (Fig. 1), suggesting that there was no significant change in *Drosophila* locomotor activity rhythm after spaceflight if flies were maintained in LD condition. However, two facts must be considered. First, our behavior test was started 48 h after landing, during which time space flown flies might have re-adapted to the Earth’s environment. Second, our activity test was conducted in LD conditions in the first five days, and since light is a strong Zeitgeber, the effect of spaceflight on activity rhythm might be masked by the light/dark cycle. Previous research on the activity rhythm of a type of desert beetle during spaceflight reported tree-running period (tau) differences caused by gravitation field changes (μg and 2G) in constant light conditions (LL and DD), but not in LD conditions, suggesting strong interference of light on the study of the effect of other Zeitgebers on the circadian clock [30]. Thus, due to the limitation in our experimental design, we cannot conclusively rule out the possibility that spaceflight might somehow affect the activity rhythm of *Drosophila*. It would be better to monitor *Drosophila* locomotor activity rhythm in constant darkness during spaceflight. Clocklab2 analysis showed that the ratio of flies that exhibited rhythmic locomotor activity was relatively low in all three groups (42.86% for space flown flies, 21% for control-1 group flies, and 50% for control-2 group flies). Considering that the tested flies were more than 30 days old, our results were consistent with published findings that older flies exhibited weaker activity rhythms than younger flies [36].

**Fig 2:**
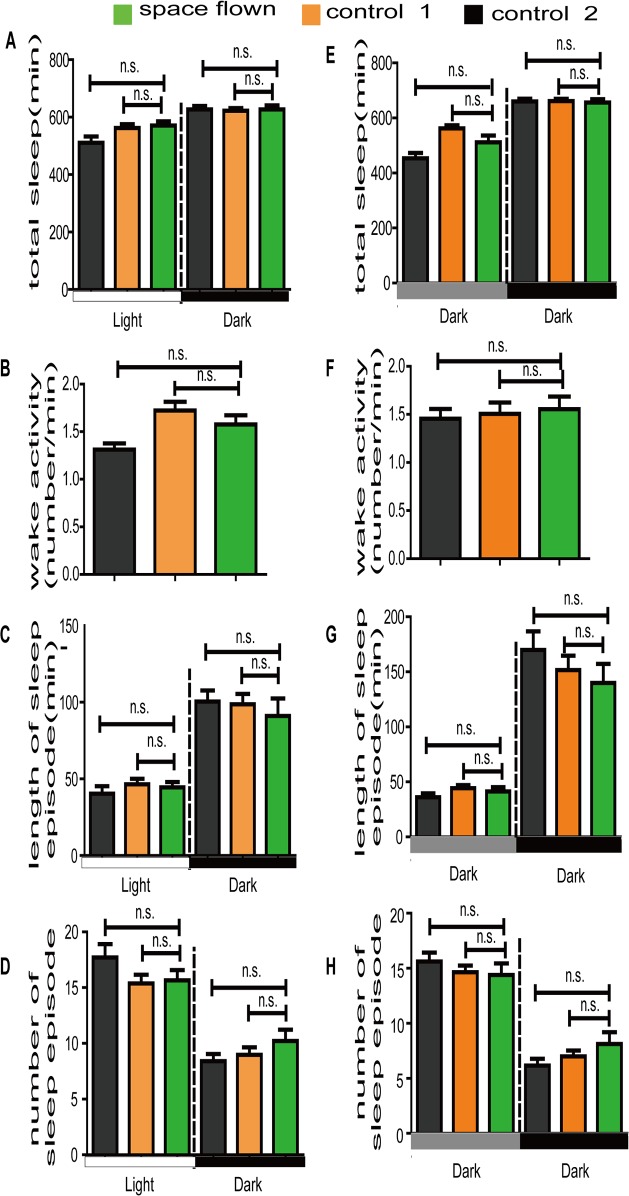
Effect of spaceflight on major sleep parameters under both LD (A-D) and DD (E-H) conditions. The average total sleep time (A, E), average length of sleep episode (C, G), and average sleep episode number (D, H) were calculated based on the *Drosophila* sleep definition. Green, orange and grey boxes represent data of space flown, control-1, and control 2 flies, respectively. White and black bars stand for daytime and nighttime, grey bar stands for subjective daytime in DD. Statistical significance was determined by two-tailed Student’s t-test with unequal variance. n.s. means no statistically significant differences. Error bars represent SEM.

We also investigated sleep patterns and three major sleep parameters in space flown flies. *Drosophila* sleep was defined as an interval of 5 min or more of behavioral immobility. We observed no differences in total sleep time among space flown flies and the two control groups in either LD or DD conditions (Figs. 2A, E and S1). Major sleep parameters, including length and number of sleep episodes, were also similar among the three groups (Fig. 2C, D, G, and H). Although there were slight differences in the length and number of nighttime sleep episodes between space flown flies and the two control flies (Fig. 2C-D, 2G-H), they were statistically insignificant. The small sample number (15 space flown flies) and the re-adaption to the Earth’s environment could be the reason for the statistical insignificance. Further research is being conducted on the effect of gravity on *Drosophila* sleep using simulated microgravity in our lab.

Unlike astronauts, who usually have a busy schedule and live in an environment with a disrupted light/dark cycle during spaceflight, the *Drosophila* did not experience intense labor pressure during spaceflight. Thus, the effect of a busy work schedule and disrupted circadian cycle on circadian rhythm and sleep could be excluded. Although no statistically significant results were obtained in our behavior test, we believe that with careful experimental design and development of improved monitoring systems [37], *Drosophila* could be a useful animal model for investigating the effect of space on animal behavior phenotypes such as sleep.

### TAG level and lipid metabolism

**Fig 3:**
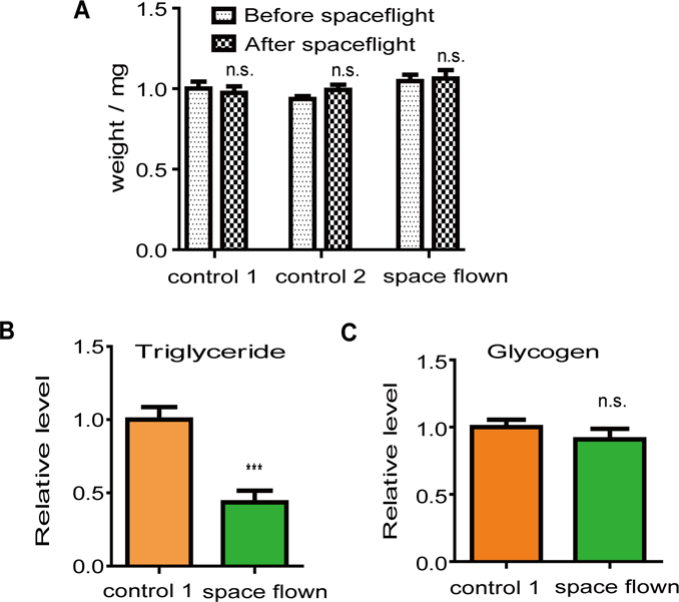
Spaceflight affected *Drosophila* lipid storage. (A), Space flown flies had similar weights as those of the two control groups. (B), Space flown flies had lower TAG levels compared with those of control-1 flies. (C), Space flown flies had similar body glycogen levels compared with those of control-1 flies. Both TAG and glycogen levels were normalized to the protein level. For each fly group, at least three independent groups of five fly bodies were analyzed. *** indicates significant differences (p<0.001). Statistical significance was determined by two-tailed Student’s t-test with unequal variance.

To study whether there was any change in *Drosophila* metabolism after spaceflight, we measured the levels of two major energy stores (TAG and glycogen) in space flown and control-1 flies during a 24-h time period. The average TAG level of space flown flies was significantly lower than the average TAG level of control-1 flies (Fig. 3B). The difference in the TAG level was not caused by fly size differences since the weight between both groups was similar (Fig. 3A). In contrast, there was no difference in glycogen levels between the two fly groups (Fig. 3C).

The decreased TAG levels in space flown flies reflected a change in lipid metabolism after the 13-day spaceflight. The metabolic change persisted two days after flies returned to Earth, suggesting that it was not a short-term effect and could not be easily compensated for by their introduction to the Earth’s environment. This result was in accordance with previous reports on human metabolism in space [38, 39], which showed that astronauts lost body fat during and after spaceflight. In astronauts, body fat loss is accompanied by decreased dietary intake during spaceflight [40], and is likely a result of negative energy balance [41]. It is possible that a similar mechanism explains the lipid metabolism change in space flown flies. However, since we did not measure *Drosophila* food intake during spaceflight in this study, we do not know whether food consumption differences account for the decreased lipid level in space flown flies. Another possibility is that increased activity during spaceflight led to the loss of energy stores in space flown flies. It has been reported that flies exhibit increased activity under a microgravity environment [42]. Although our locomotor activity results showed that space flown flies were not more active than ground controls after they returned to Earth, they were monitored 48 h after landing, so we cannot rule out the possibility that *Drosophila* locomotor activity was increased during spaceflight, and the decreased TAG levels were caused by increased activity during spaceflight.

### Lifespan

**Fig 4:**
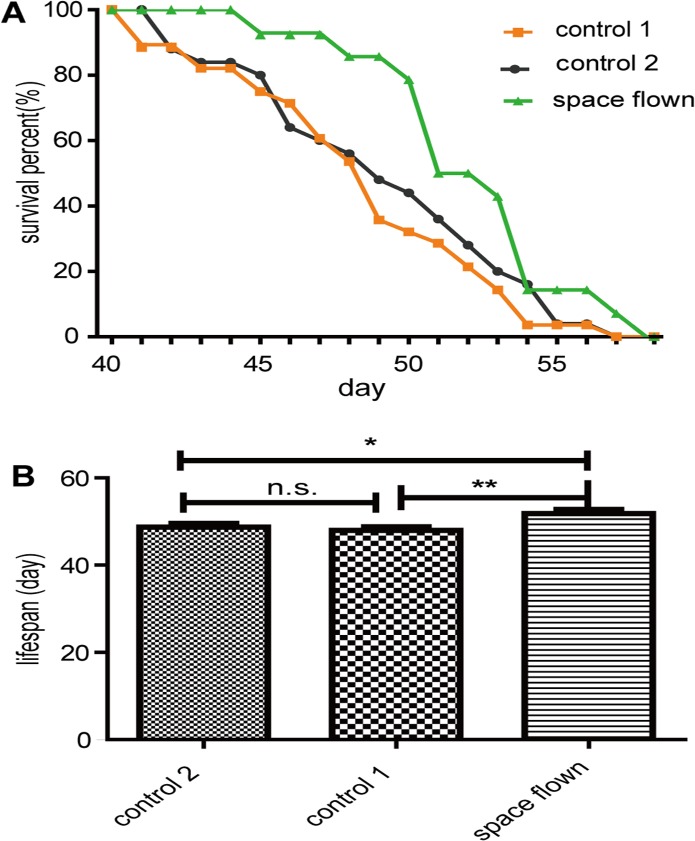
Spaceflight affected *Drosophila* lifespan. Space flown flies had significantly extended lifespan. (A): Survival curve for space flown, control-1 and control-2 flies; (B): Average lifespan comparison. Error bars represent SEM. *** indicates significant differences (p<0.001). Statistical significance was determined by two-tailed Student’s t-test with unequal variance.

We also examined changes in *Drosophila* lifespan after spaceflight. Fig. 4 presents the survival curves and average lifespan of the space flown and two control groups. Results showed that space flown flies lived slightly longer than the two control groups. The average lifespan of space flown flies was 51.82 ±0.93 days, while the average lifespan of control-1 and control-2 flies was 47.96 ±0.82 and 48.71 ±0.88 days, respectively. Although the small number of tested animals reduced the credibility of the results and further repeat experiments are necessary, the lifespan differences between space flown and control flies were statistically significant, indicating there might be a weak effect of spaceflight on *Drosophila* lifespan.

It was reported that *Drosophila* lifespan can be regulated by both the insulin dependent pathway and insulin independent dietary restriction [43]. Considering that no mRNA level changes in the insulin-pathway related genes were detected by microarray, except for *ilp3*, and metabolism tests showed that TAG levels decreased in space flown flies, we hypothesized that the TAG level decrease indicated a dietary restriction during spaceflight, which, in turn, caused a lifespan extension through insulin independent mechanisms. However, further evidence is needed to confirm this hypothesis.

How spaceflight affects aging is still largely unknown. It was once widely accepted that the space environment accelerates the aging process [44, 45]. However, there has been little evidence that directly proves whether the space environment influences animal lifespan. Recent research reported that genes involved in nematode lifespan extension were up-regulated after spaceflight [46], indicating that spaceflight might extend the life of *Caenorhabditis elegans*. Our results, if they could be repeated, also suggested that spaceflight could extend *Drosophila* lifespan. Thus, the opinion that space accelerates the aging process is becoming controversial and needs more supporting evidence. With the increasing duration of spaceflight missions, such as human exploration of other planets or colonization of the moon, studies on the effect of the space environment on lifespan has become of increasing medical importance and will attract growing attention in the future.

### Microarray analysis

We used microarray to profile gene expression in the heads of space flown flies as well as the two control groups. Samples from six time points during a 24-h period under LD conditions were hybridized with the Affymetrix GeneChip *Drosophila* Genome Array 2.0 containing 18,500 probe sets. Each sample was hybridized to two chips to test reproducibility. The mean hybridization signal strength and the SEM for each probe set were calculated from the duplicate hybridizations. S1 Table shows the expression levels of the 4483 genes obtained from the microarray experiment.

In the experimental design, we started sample collection for RNA extraction almost 50 h after landing. During this period of time flies underwent some re-adaptation to the Earth’s gravitational environment, which made it difficult to evaluate the role of spaceflight on gene expression changes. However, many previous studies have found that some physiological effects of spaceflight might last much longer. For example, post flight swelling and elevation of human calf muscle transverse relaxation time persisted for several weeks after flight [47], and at two weeks post recovery, the hamstrings and intrinsic lower back muscles were still below baseline [48]. Studies on *Drosophila* immune response also found that the immune system of space flown flies function more effectively than that of ground control flies 3 days post landing [34]. Furthermore, the lipid store differences we detected also suggested that some physiological changes in space flown flies could persist, even 48h after landing. Hence, we expected that our microarray analysis would still detect some long-term effects of spaceflight on *Drosophila* gene expression.

### Effect of sampling time on the analysis of gene expression differences

**Fig 5:**
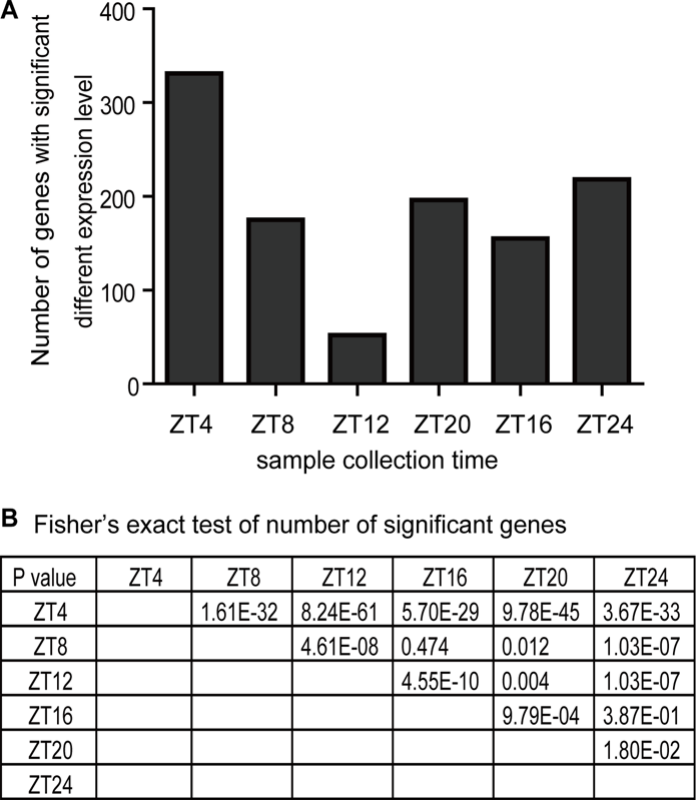
Effect of sampling time on the analysis of gene expression differences between space flown and control flies. (A) Gene expression levels in head samples collected at six time points during a 24-h period under LD conditions were compared between space flown flies and the two control groups. The number of genes that exhibited significant expression level change (>1.5 fold) at each time point were recorded. (B) Fisher’s exact test was used to determine the statistical significance of the differences between the data from every two time points. P<0.05 indicates significant differences.

It is well known that many genes exhibit a circadian rhythmic expression pattern in *Drosophila*, thus gene expression comparison between two fly samples could vary according to the different sampling times over a 24-h time period under LD conditions. Here we searched for genes with significant expression level changes, defined as exhibiting more than a 1.5-fold change between space flown and control samples, at different time points, and then counted the number of genes with significant expression level changes at each time point (Fig. 5). The numbers of genes with significant expression level changes varied according to the different sampling times. At ZT4, for example, there were 330 genes with significant expression level change (up 127; down 203). At ZT12, however, the number decreased to only 52 genes (up 32; down 20). Our results showed that sampling time can greatly affect gene expression analysis results, suggesting the necessity to follow an appropriate sampling procedure in the investigation of the effect of spaceflight on gene expression. Not only should space flown samples be collected as early as possible after they return to Earth, but samples should also be collected at either the same Zeitgeber time or multiple time points during a 24-h period. Otherwise, it is difficult to obtain repeatable data even if all other experimental conditions are strictly controlled.

We determined two hypotheses that may explain the effect of sampling time on the analysis of gene expression differences. Firstly, the biological effects of spaceflight might be cycled during the 24-h period. As we know, the circadian clock regulates various physiological functions, and the activity of a clock-controlled physiological process will likely exhibit a circadian rhythmic pattern during spaceflight. The responses to space between the peak and trough times of the activity curve might be different, which might, in turn, result in expression level change differences of genes involved in such a physiological process. Secondly, spaceflight might cause phase changes in some physiological rhythms. Thus, genes involved in these processes might also experience phase changes in space flown samples. As a result, the expression of these genes at certain time points might show significant differences between space flown and control samples. It is unlikely that the differences were caused by the alleviation of the spaceflight effect after landing, since the number of genes with significant expression level change increased again after reaching the trough at ZT12. However, as the number of genes with significant expression level change at the last sampling time point were less than that at the first time point, it is still possible that the differences in time since landing could also contribute to this phenomenon.

### Spaceflight affected *Drosophila* gene expression

As shown above, gene expression level differences could vary according to the different sampling time in LD conditions, and measuring expression differences at only one single time point was unsatisfactory. Thus, we combined the gene expression data of all six time points and calculated the average expression level of each gene obtained from the microarray results. We used both statistical (unpaired Student’s *t*-test, P<0.05) and fold change (more than 1.5-fold) thresholds as criteria for selecting genes with significant level change. We identified 54 genes with significant level change between space flown and control-1 flies (37 genes, up; 17 genes, down) (S2 Table) and 55 genes between space flown and control-2 flies (30 genes, up; 25 genes, down) (S3 Table). To reduce false positives, we next only selected genes that appeared on both lists, which reduced the number to 12. We listed these as genes with significant mRNA level change after spaceflight (Table 1).

**Table 1:** Genes with significantly changed expression level after spaceflight.

Representative Public ID	Gene Symbol	Spaceflight /control-1	Spaceflight /control-2	Function
**CG18290-RA**	Act87E	1.58	1.68	Structural constituent of cytos- keleton, cytoskeleton organization
**CG31149-RA**	CG42613	0.65	0.51	Low-density lipoprotein (LDL) receptor class A repeat
**CG4784-RA**	Cpr72Ec	1.94	1.91	Structural constituent of chitin-based cuticle
**CG14167-RA**	Ilp3	1.62	2.45	Insulin receptor binding, female mating behavior
**CG17461-RA**	Kif3C	0.65	0.53	Microtubule motor activity, neuron projection morphogenesis
**CG30179-RA**	Mlp60A	1.71	1.55	Zinc ion binding, muscle tissue development
**CG2380-RA**	NfI	0.63	0.62	Sequence-specific DNA binding transcription factor activity
**CG10806-RB**	Nha1	1.70	2.10	Sodium: hydrogen antiporter activity
**CG8462-RA**	Obp56e	1.75	2.26	Odorant binding,multi cellular org- anism reproduction
**CG1748-RA**	RhoGAP102A	0.63	0.56	Rho GTPase activator activity
**CT36054**	SK	1.84	1.76	Photoreceptor activity,regulation of membrane potential in photo- receptor cell
**CG15427-RC**	tutl	1.66	1.79	Synaptic target recognition,regulation of dendrite morphogen- esis,axon guidance and defascicu- lation,larval and adult behavior regulation

We noticed a small overlap between the results obtained using the different controls. It is worth noting, however, that control-2 flies were maintained under ideal conditions in our lab incubator, while control-1 flies were maintained in similar conditions as the space flown flies. Furthermore, control-1 flies also underwent a simulated launching process and the same ground travel experience as space flown flies. Thus, the small overlap between the gene expression data between the two control groups might reflect the effect of stress on gene expression.

Compared with other microarray reports investigating the space effect on gene expression [33, 49], we detected far fewer genes with significant mRNA level change after spaceflight. This could be due to different experimental design. Firstly, we compared the average mRNA level of data from six time points in a 24-h period, not just data at a specific time point. As discussed above, our sampling design can exclude of sampling time on gene expression analysis. Secondly, the short-term effect of spaceflight on gene expression was excluded in our study. Due to technical limitations, sample collection began 48 h after landing, much later than in the other reports. Thus, the expression level of many genes may have already recovered before we commenced sample collection. Thirdly, unlike brain samples, there are many tissue and cell types in *Drosophila* heads, so some spaceflight effects in certain tissues or cells might be masked. Finally, for some reason, we only obtained reliable gene expression data for 4483 genes from our microarray hybridization experiment, which also contributed to the shorter list of genes affected by spaceflight.

Functional analysis of the 12 genes revealed one gene, *ilp3*, that might be involved in *Drosophila* metabolism and lifespan regulation [50]. *Drosophila* regulates energy metabolism using a system similar to the insulin/glucagon system used in mammals [51]. There are seven insulin-like peptides (ILPs) in *Drosophila*, among them *ilp2* has the most potent function in regulating lifespan and metabolism [52]. Although *ilp3* is not directly involved in lifespan regulation, its expression is closely related to *ilp2* mRNA level. Knock-down of *ilp2* has been reported to result in the up-regulation of *ilp3* via direct autocrine regulation through the insulin signaling pathway [50], indicating that the increase in *ilp3* mRNA level might be caused by a compensation effect of decreased *ilp2*. Although we did not detect any mRNA level change of other ILPs or genes involved in the insulin signal transduction pathway, it is possible that changes in insulin related genes in certain cell types might be masked as we used a mixture of various cell types as the source of our mRNA sample for microarray. Alternatively, since the reduction of *ilp2* cannot lead to lifespan extension, the *ilp3* mRNA change in space flown flies might have nothing to do with the low lipid level and longer lifespan observed. Further research is needed to unveil the molecular mechanism and genes involved.

### Major clock genes were unaffected after spaceflight

To determine whether there was any difference in the *Drosophila* circadian clock after spaceflight, we investigated the rhythmic expression pattern of major clock genes (*per*,*tim*,*vri*, and *cry*) in space flown and control flies. As shown in S2 Fig, all four clock genes exhibited rhythmic patterns in both space flown and control flies at a similar phase, suggesting that the function of the circadian oscillator in fly heads remained normal in LD conditions 48 h after landing. However, since 48 h is enough time for the re-adaption of the *Drosophila* circadian clock from the space to Earth environment, we cannot exclude the possibility that the *Drosophila* circadian clock might be changed during spaceflight. Future microarray analysis using samples collected during spaceflight is necessary to make such conclusions.

### Identification of cyclic genes

Due to the insufficient number of space flown flies, we only collected samples at six time points in a 24-h period. However, most software used for detecting circadian expressed genes requires at least 12 samples in a 24- to 48-h period. Thus, we needed special analysis software to search circadian expressed genes from our microarray database. We chose STEM software developed for the analysis of short time series gene expression data [35]. STEM can identify statistically significant temporal expression profiles and associated genes. In our analysis, STEM first selected 25 distinct and representative temporal expression profiles as model profiles independent of the data; then assigned each of the 4483 genes passing the filtering criteria to the model profile that most closely matched the gene’s expression profile; and finally determined which model profiles had significantly more assigned genes compared to the average number assigned to the model profile, with significant model profiles highlighted in color. The analysis results are presented in S3A-S3C Fig.

STEM could not define the circadian expression profiles, so we established our own criterion and defined circadian expression profiles as profiles with expression patterns close to a cosine wave. Using this criterion, we selected six model circadian expression profiles: profile 0, 4, 8, 16, 22, and 24. For space flown flies, only one circadian expression profile (profile 16) belonged to the significant model profiles, but more circadian expression profiles belonged to significant model profiles for the two control groups (S3A-S3C Fig.). Genes grouped into circadian expression model profiles of the space flown flies and the two control groups are listed in S4 Table and defined as circadian expressed genes. In total, there were 104, 90 and 335 circadian expressed genes in space flown, control-1 and control-2 flies, respectively.

From the circadian expressed gene lists, most circadian expressed genes in space flown flies were not on the circadian expressed gene lists of the two control groups. Only 21 genes were circadian expressed in all three samples (S3D Fig.), including the four circadian clock genes investigated above. Since circadian expressed genes are generally accepted as output genes of the circadian clock system, our results indicated that although the *Drosophila* circadian core oscillator was not changed after spaceflight, there were changes in the circadian clock output pathway after spaceflight.

We also investigated the phase differences of genes that showed circadian expression pattern in both space flown flies and at least one control group, which showed that some genes exhibited phase changes in space flown flies. For example, the peak level of *cpr60d* mRNA in space flown flies was at ZT4, while in the two control groups, the peak level appeared at ZT20 and ZT16, respectively, suggesting an 8 to 12 h phase change (S4A Fig.). Furthermore, *mus209* exhibited circadian expression pattern in both space flown and control-1 flies but had a 4 to 8 h phase advance in space flown flies (S4B Fig.). Since the four major circadian clock genes in fly heads did not show any phase shifts, it was unlikely that the phase of the *Drosophila* central clock system was changed after spaceflight. The circadian clock exists not only in the brain, but also in many other peripheral tissues [53–55], and some environmental stimuli can only entrain the peripheral clock with no effect on the central clock [56]. Thus, it is possible that during spaceflight certain peripheral clocks experienced a phase change, and in turn caused the phase changes of some circadian output genes highly expressed in these peripheral tissues.

In this paper, we tested the effect of spaceflight on the circadian output pathway using two different assays. The locomotor activity rhythm of space flown flies was normal, suggesting that either the effect of spaceflight on *Drosophila* locomotor activity rhythm was too weak to be detected, or locomotor activity rhythm could quickly readapt to the Earth’s environment. However, gene expression analysis detected rhythmic changes of some circadian output genes in space flown flies, indicating that assays at the molecular level were more sensitive than behavioral assays in detecting the effect of spaceflight on the circadian clock output pathway. However, with our current experimental design and results, we could not determine whether such changes in the circadian output pathway were caused directly by spaceflight or indirectly by the result of physiological changes after spaceflight. Finally, although we divided our samples into two pools before RNA extraction, this was not a biological repetition. Repeat microarray experimentation is needed to verify our results.

### QPCR

**Fig 6:**
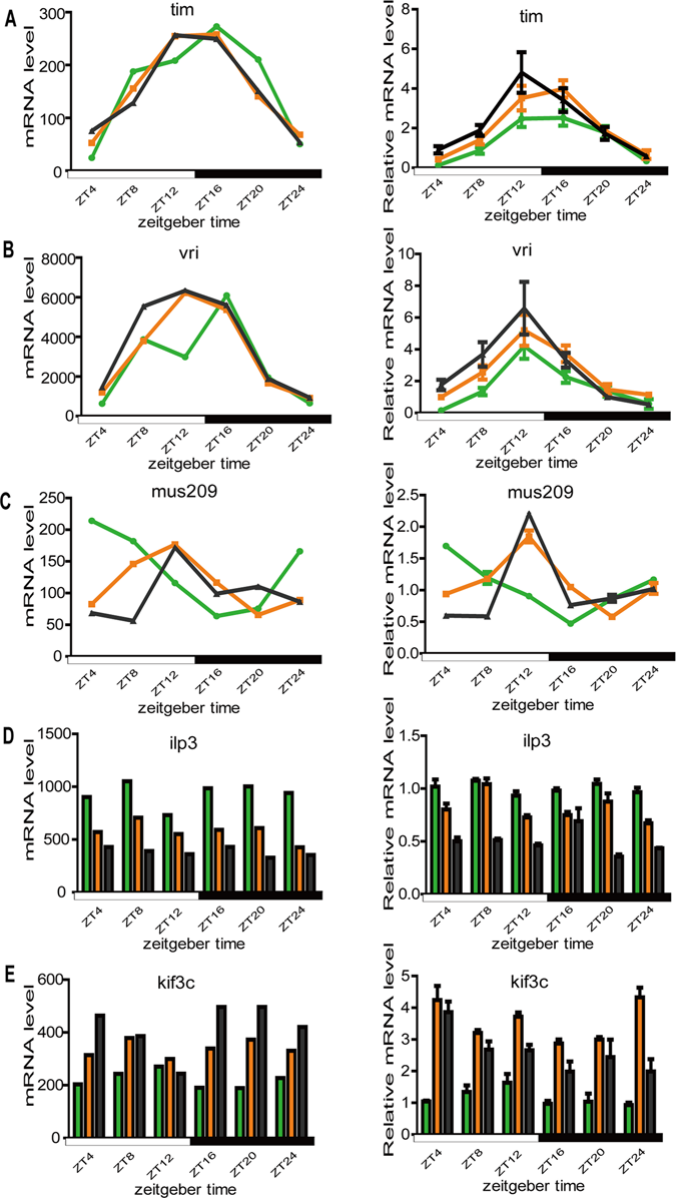
Validation of microarray result for selected genes by QPCR. (A) *tim*; (B) *vri*; (C) *mus209*; (D) *ilp3*; (E) *kif3c*. For each gene, the left plot is the microarray result and the right is the QPCR result. For each graph, the green, orange, and blank lines or histograms represent the space flown group, control-1 group and control-2 group, respectively.

Since microarray is a semi-quantitative tool for examining expression differences, we performed QPCR to confirm the observed gene expression differences and phase changes in rhythmic expressed genes from our microarray analysis. Two major circadian clock genes (*tim* and *vri*), two genes with changed expression levels after spaceflight (*ilp3* and *kif3c*), and one rhythmic gene with a shifted phase after spaceflight (*mus209*) were tested by QPCR. Generally, the QPCR results closely matched the microarray results (Fig. 6): the expression of circadian clock genes were not affected by spaceflight; *ilp3* mRNA expression level was up-regulated in spaceflight group samples, while *kif3c* mRNA expression level was down-regulated; and, the phase of *mus209* expression rhythm exhibited an 8 h advance according to its peak time. These results supported the validity of our microarray analysis.

## Supporting Information

S1 Fig Spaceflight had no effect on *Drosophila* sleep profile. Space flown flies exhibited similar sleep profiles as the two control groups under both LD (A) and DD (B) conditions. *Drosophila* sleep was defined as an interval of 5 min or more of behavioral immobility. Sleep profiles were analyzed using Pysolo 0.9 software. White and black bars stand for daytime and nighttime, grey bar stands for subjective daytime in DD. Error bars represent SEM.
(TIF)

S2 Fig Gene expression curves from microarray data showed that the rhythmic expression of major circadian clock genes did not change after spaceflight. (A) *vri*; (B) *tim*; (C) *per*; (D) *cry*.
(TIF)

S3 Fig Model profile overview of gene expression analysis at different time points under LD conditions. (A), space flown flies; (B), control-1 flies; and (C) control-2 flies. The data was sampled at six time points: ZT0, ZT4, ZT8, ZT12, ZT16, and ZT20. The colored profiles had a statistically significant number of genes assigned. We defined six profiles (profile 0, 4, 8, 16, 22, 24) as circadian expression profiles, and genes assigned into these six profiles were identified as circadian expressed genes. (D) Rhythmic expression genes of space flown flies and two control groups. The blue, red and green circles represent the rhythmic expressed genes of space flown, control-1, and control-2 flies, respectively. The numbers in the non-overlapping part of the circle represent the numbers of genes that showed circadian expression patterns only in one fly group. The numbers in the overlapping part of different circles represent the numbers of genes that showed circadian expression patterns in more than two fly groups.
(TIF)

S4 Fig Phase change of some circadian expressed genes after spaceflight. (A) *cpr60d* (B) *mus209*.
(TIF)

S1 Table Microarray results of 4483 genes of space flown flies and the two control groups at six time points. (XLS)

S2 Table List of genes that showed significantly different expression levels between space flown and control-1 flies. (XLS)

S3 Table List of genes that showed significantly different expression levels between space flown and control-2 flies. (XLS)

S4 Table List of genes of space flown flies and the two control groups that belong to the six circadian expression profiles. (XLS)
