# Correction: Effect of Spaceflight on the Circadian Rhythm, Lifespan and Gene Expression of *Drosophila melanogaster*

**DOI:** 10.1371/journal.pone.0139758

**Published:** 2015-10-08

**Authors:** Lingling Ma, Jun Ma, Kanyan Xu

The following information is missing from the Funding section: “The authors received internal funding for this project from the China Aerospace Science and Technology Corporation (CAST): http://english.spacechina.com/n16421/index.html. The funder had no role in study design, data collection and analysis, decision to publish, or preparation of the manuscript.”

The authors have also provided an updated Competing Interests section: “The authors received internal funding for this project from the China Aerospace Science and Technology Corporation (CAST): http://english.spacechina.com/n16421/index.html. This does not alter the authors’ adherence to PLOS ONE policies on sharing data and materials.”
