# Supplementary figures and images for: Effect of Spaceflight on the Circadian Rhythm, Lifespan and Gene Expression of Drosophila melanogaster
Source: PLoS One. 2015 Mar 23;10(3):e0121600. doi: 10.1371/journal.pone.0121600 (PMC4370389; doi:10.1371/journal.pone.0121600)

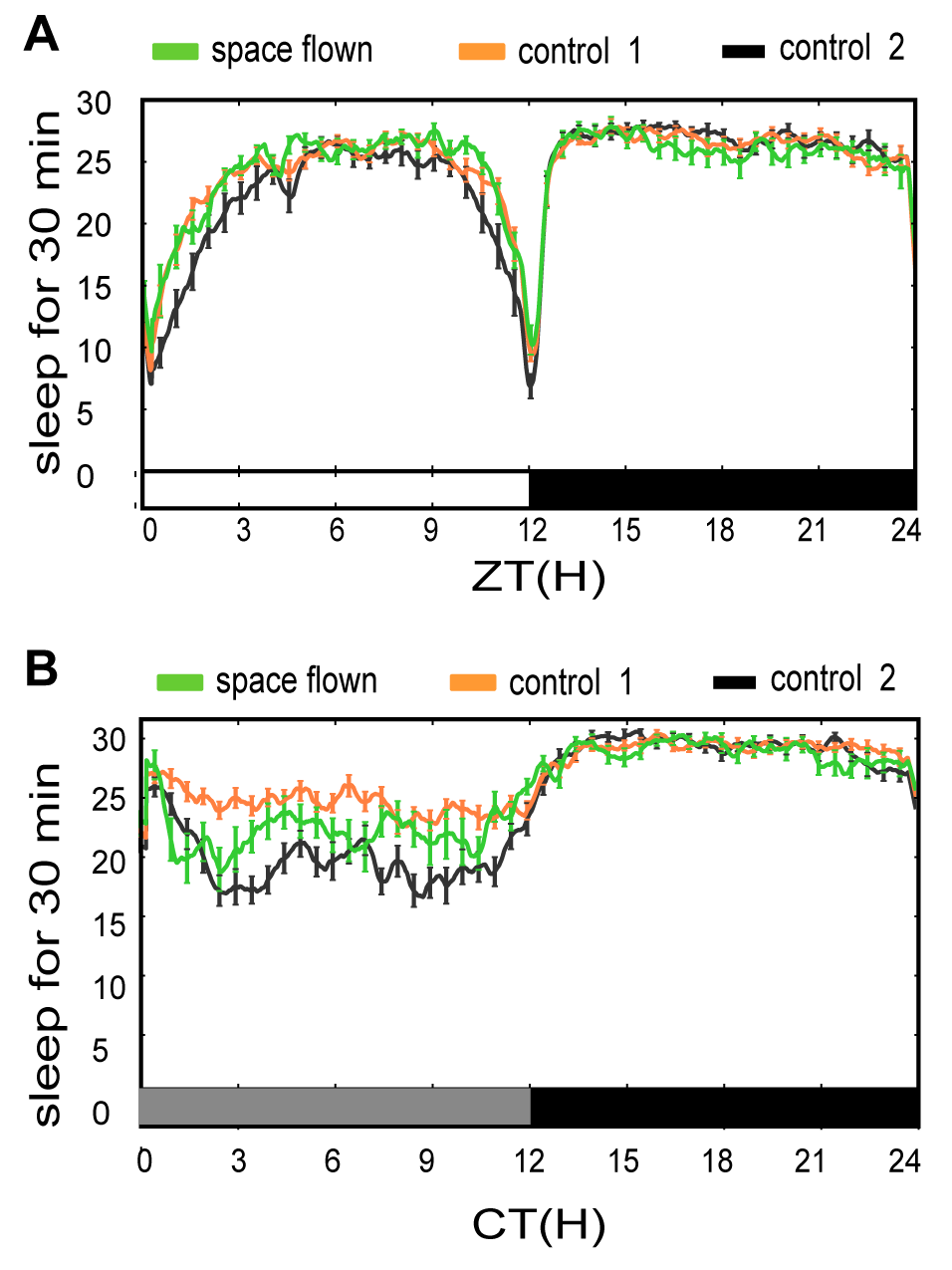

Supplement: S1 Fig — Space flown flies exhibited similar sleep profiles as the two control groups under both LD (A) and DD (B) conditions. Drosophila sleep was defined as an interval of 5 min or more of behavioral immobility. Sleep profiles were analyzed using Pysolo 0.9 software. White and black bars stand for daytime and nighttime, grey bar stands for subjective daytime in DD. Error bars represent SEM. (TIF) [file pone.0121600.s001.tif]

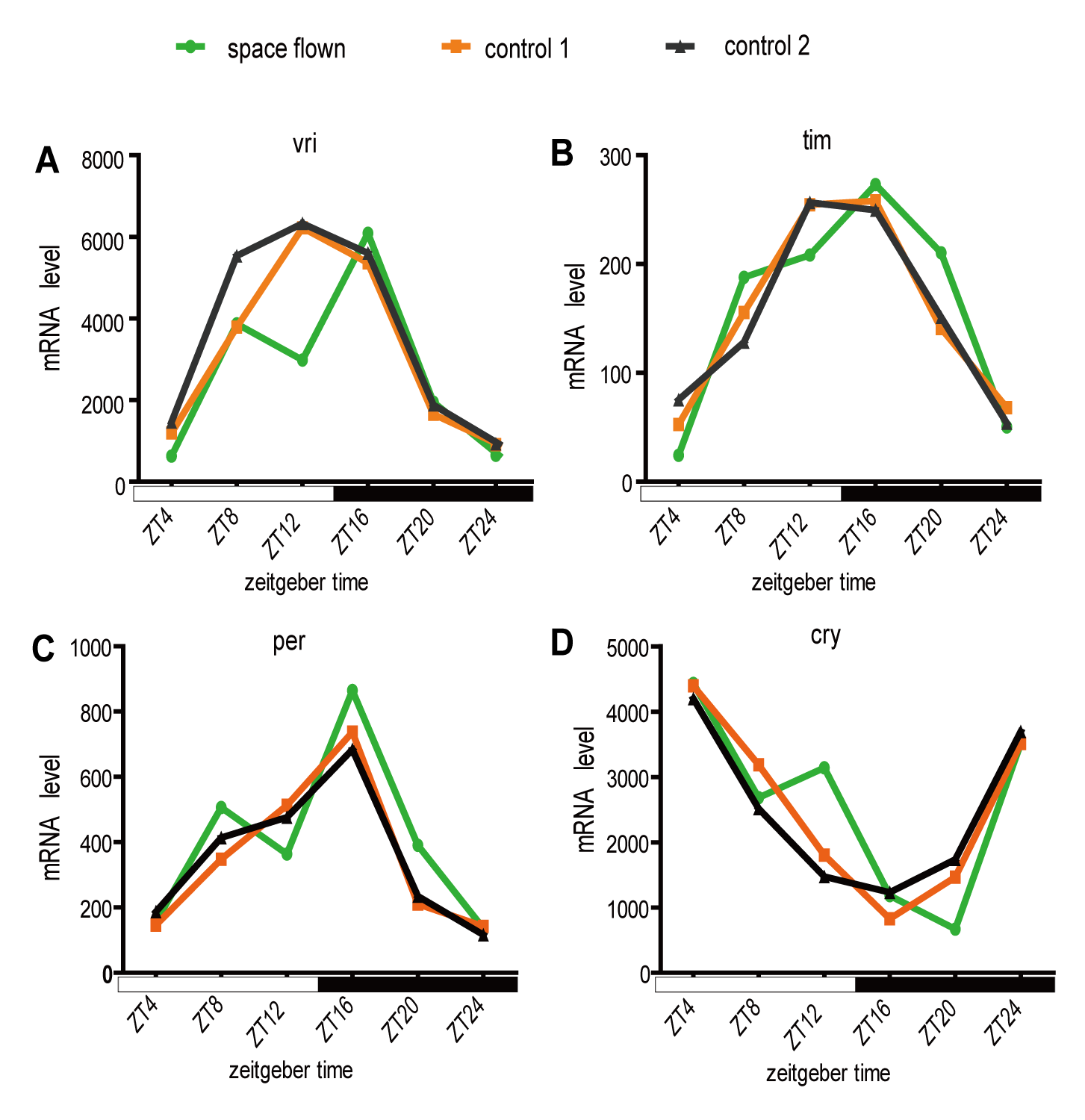

Supplement: S2 Fig — (A) vri; (B) tim; (C) per; (D) cry. (TIF) [file pone.0121600.s002.tif]

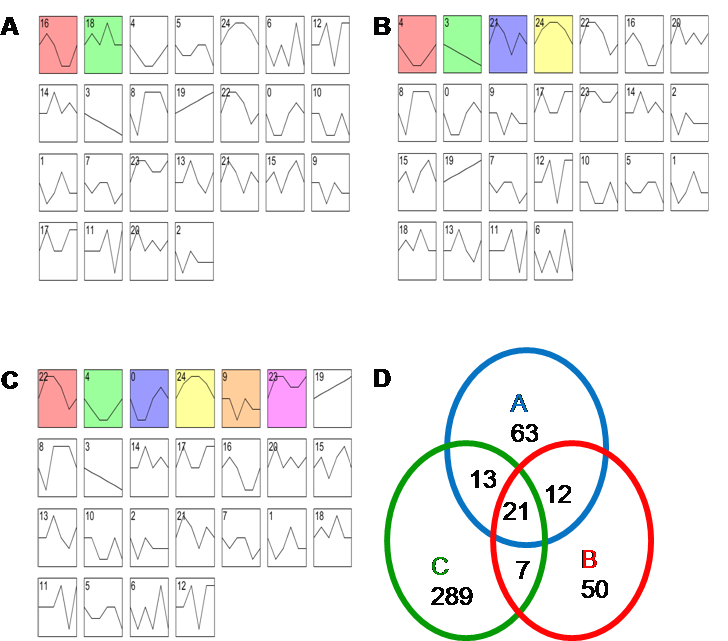

Supplement: S3 Fig — (A), space flown flies; (B), control-1 flies; and (C) control-2 flies. The data was sampled at six time points: ZT0, ZT4, ZT8, ZT12, ZT16, and ZT20. The colored profiles had a statistically significant number of genes assigned. We defined six profiles (profile 0, 4, 8, 16, 22, 24) as circadian expression profiles, and genes assigned into these six profiles were identified as circadian expressed genes. (D) Rhythmic expression genes of space flown flies and two control groups. The blue, red and green circles represent the rhythmic expressed genes of space flown, control-1, and control-2 flies, respectively. The numbers in the non-overlapping part of the circle represent the numbers of genes that showed circadian expression patterns only in one fly group. The numbers in the overlapping part of different circles represent the numbers of genes that showed circadian expression patterns in more than two fly groups. (TIF) [file pone.0121600.s003.tif]

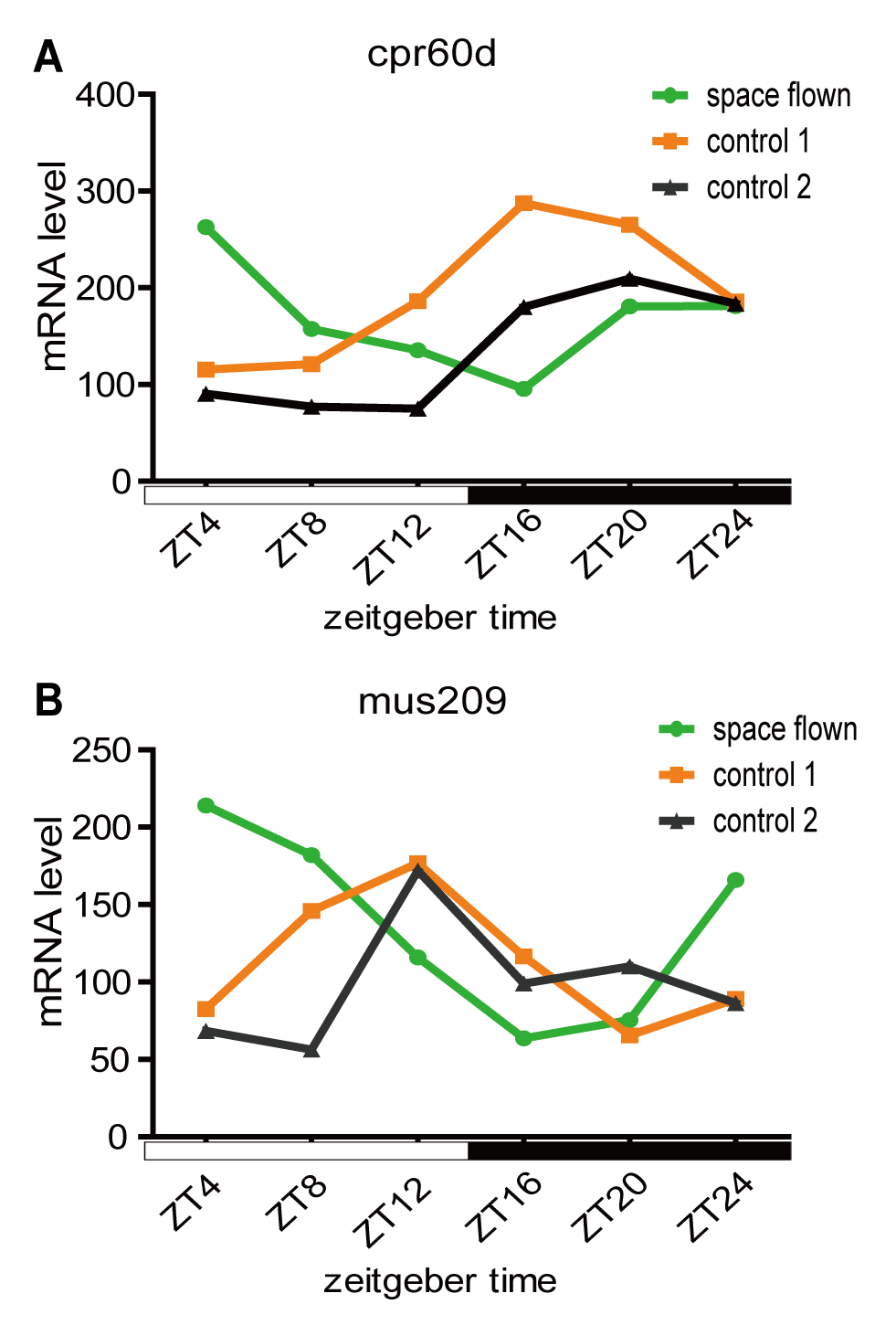

Supplement: S4 Fig — (A) cpr60d (B) mus209. (TIF) [file pone.0121600.s004.tif]
